# Probiotics and virulent human rotavirus modulate the transplanted human gut microbiota in gnotobiotic pigs

**DOI:** 10.1186/s13099-014-0039-8

**Published:** 2014-09-09

**Authors:** Husen Zhang, Haifeng Wang, Megan Shepherd, Ke Wen, Guohua Li, Xingdong Yang, Jacob Kocher, Ernawati Giri-Rachman, Allan Dickerman, Robert Settlage, Lijuan Yuan

**Affiliations:** 1 Department of Civil and Environmental Engineering, Virginia Polytechnic Institute and State University, Blacksburg 24061, VA, USA; 2 Department of Biomedical Sciences and Pathobiology, Virginia-Maryland Regional College of Veterinary Medicine, Virginia Polytechnic Institute and State University, Blacksburg 24061, VA, USA; 3 Department of Large Animal Clinical Sciences, Virginia-Maryland Regional College of Veterinary Medicine, Virginia Polytechnic Institute and State University, Blacksburg 24061, VA, USA; 4 Virginia Bioinformatics Institute, Blacksburg 24061, VA, USA; 5 College of Animal Science & Technology, Zhejiang A & F University, Zhejiang Province, China

**Keywords:** Microbiota, Gnotobiotic pigs, Rotavirus, Vaccine, Lactobacillus, Probiotics

## Abstract

We generated a neonatal pig model with human infant gut microbiota (HGM) to study the effect of a probiotic on the composition of the transplanted microbiota following rotavirus vaccination and challenge. All the HGM-transplanted pigs received two doses of an oral attenuated rotavirus vaccine. The gut microbiota of vaccinated pigs were investigated for effects of *Lactobacillus rhamnosus* GG (LGG) supplement and homotypic virulent human rotavirus (HRV) challenge. High-throughput sequencing of V4 region of 16S rRNA genes demonstrated that HGM-transplanted pigs carried microbiota similar to that of the C-section delivered baby. Firmicutes and Proteobacteria represented over 98% of total bacteria in the human donor and the recipient pigs. HRV challenge caused a phylum-level shift from Firmicutes to Proteobacteria. LGG supplement prevented the changes in microbial communities caused by HRV challenge. In particular, members of *Enterococcus* in LGG-supplemented pigs were kept at the baseline level, while they were enriched in HRV challenged pigs. Taken together, our results suggested that HGM pigs are valuable for testing the microbiota’s response to probiotic interventions for treating infantile HRV infection.

## 1 Introduction

Humanized microbiota models, such as germ-free animals transplanted with human feces, are valuable for studying microbiota composition change due to external factors by minimizing confounding variables [1],[2]. Gnotobiotic (Gn) pigs provide an excellent model for isolating microbiota as an environmental factor in disease models [3] because pigs and human share high genome homology (98%), similar intestinal anatomy, physiology, immune systems, nutritional requirements, and food transit times [4]–[6]. Both pigs and human are also susceptible to human rotavirus Wa strain (genotype G1P [7]) infection and disease [8].

Human rotavirus (HRV) infection is the leading cause of gastroenteritis in infants and children, especially in developing countries [7]. The burden is exacerbated for infants and young children in the developing world, because many of them have a weak immune response to oral rotavirus vaccines and the protective efficacies of the vaccines are lower compared to that in the developed world [9]. The gut microbiota’s response to rotavirus infection has not been systematically investigated; despite that, microbiota disruption (intestinal dysbiosis) may be a risk factor for long-term adverse effects. Rotavirus infection in humans was found to be associated with an increase in *Bacteroides fragilis* and decreased *B. vulgatus* and *B. stercoris* in a clone-library study [10]. However, a comprehensive analysis is lacking for the effect of therapeutic interventions such as vaccination and probiotics on rotavirus-infected gut microbiota.

Orally administered probiotic bacterium *Lactobacillus rhamnosus* GG (LGG) has been tested in numerous clinical trials to prevent or shorten rotavirus-induced diarrhea [11],[12]. Supplementation with LGG for 4 weeks after acute rotavirus infection reduced intestinal permeability in children with rotavirus diarrhea, reduced the number of subsequent diarrheal episodes and increased IgG antibody response [13]. Lactobacilli have also been implicated in lipid metabolism due to their bile salt hydrolase activities [14]. We hypothesized that the beneficial effects of LGG against rotavirus-related diarrhea may be a result of modulating the intestinal microbiota towards a healthier profile.

We aimed to determine how rotavirus infection affects the human gut microbiota inoculated in Gn pigs and whether probiotic LGG can prevent the disruption of the microbiota. We transplanted human infant fecal microbiota to newborn Gn pigs. The pigs were or were not treated with a daily dose of LGG for 2 weeks, vaccinated with an oral attenuated HRV vaccine, and subsequently challenged or not challenged with virulent HRV. We investigated: (a) how efficiently different bacterial species in the human gut microbiota (HGM) colonize neonatal germ-free pigs, (b) the associative changes in the microbiota in response to HRV challenge, and (c) whether LGG, HRV and interactions between LGG and HRV have effects on the gut microbial community structure.

## 2 Materials and methods

### 2.1 Ethics statement

All animal experiments were performed in strict accordance with federal and university guidelines. Specifically, we adhered to the recommendations in the Guide for the Care and Use of Laboratory Animals of the National Institutes of Health and the American Veterinary Medical Association Guidelines on Euthanasia. The animal protocol was approved by the Institutional Animal Care and Use Committee at Virginia Tech (Protocol# 10-168-CVM and 13-187-CVM). Ethical Committee approval was received from Virginia Tech Institutional Review Board for the newborn human stool sample collection (IRB number 11–1049).

### 2.2 Transplantation of HGM into Gn pigs

Gn pigs were derived by hysterectomy from near-term sows (Landrace and Large White crossbred) and maintained in germ-free isolator units [8],[15]. Pigs of the same treatment groups were housed in individual 4-place isolators. The pigs were fed ultra-high-temperature sterilized milk (Hershey) throughout the experiment.

Multiple stool samples from a cesarean-section delivered, exclusively breast-fed healthy infant at 17–23 days of age were collected and made into an inoculum pool to generate HGM pigs. Briefly, daily collected fresh stool was diluted 20-fold in sterile pre-reduced PBS (pH 7.2) and glycerol (15% by volume) and stored at −80°C under an atmosphere of nitrogen. A week-long multiple stool samples were pooled and homogenized for use in the entire course of the experiment. Prior to inoculation, the human stool was screened for pathogens as previously described [16]. The screening showed no hemolytic activity. Next-generation sequencing at Viral Diagnostics and Discovery Center at UCSF showed no known viruses in the sample. Pigs were orally inoculated with the human stool inoculum (1 ml of 5% stool suspension in PBS) once daily starting at 12 hours after birth for three days to establish the HGM in Gn pigs. The timing of HGM inoculation is to mimic the natural microbial colonization of the newborn’s gut (within hours after birth).

### 2.3 Inoculation of Gn pigs with attenuated HRV vaccine, virulent HRV and probiotics (LGG)

The cell-culture adapted attenuated HRV Wa strain (G1P1A [7]) was used as the vaccine at a dose of 5 × 10^7^ fluorescent focus forming units (FFU) [8]. The virulent HRV Wa strain was passaged through Gn pigs and the pooled intestinal contents were used for challenge of Gn pigs at a dose of ~10^5^ FFU [17]. The virus titer was determined by using cell culture immunofluorescence (CCIF) assay and was expressed as FFU/ml as described previously [18]. Probiotic LGG (ATCC# 53103) was propagated in Lactobacilli MRS broth (Weber, Hamilton, NJ, USA). LGG inoculums were prepared and titrated as previously described [19].

All the HGM pigs received two dose of the oral attenuated HRV vaccine at 5 and 15 days of age. The purpose of the vaccination is to study the effects of LGG in enhancing the immunogenicity of rotavirus vaccines in the HGM pigs, which was the objective of another concurrent study [16]. The HGM-transplanted and vaccinated pigs were divided into four groups: (a) no LGG feeding, no virulent HRV challenge (−LGG-HRV, n = 4), (b) no LGG feeding, with HRV challenge (−LGG + HRV, n = 4), (c) with LGG feeding, no HRV challenge (+LGG-HRV, n = 4), and (d) with both LGG feeding and HRV challenge (+LGG + HRV, n = 3, one pig was euthanized prior to the scheduled time due to health problems). Daily LGG feeding started at 3 days of age for 14 days (3–16 days of age) with 10-fold incremental LGG dose increase every day (from 10^3^ to 10^9^ CFU/dose) as previously described [19]. LGG was administrated in 3 ml of 0.1% peptone water. Non-LGG fed pigs were given 3 ml of 0.1% peptone water but no LGG. Pigs in the + HRV groups were challenged with the virulent HRV at post-attenuated HRV inoculation day (PID) 28. Pigs were euthanized at PID 28 before challenge (−LGG-HRV and + LGG-HRV groups) or at post-challenged day (PCD) 7 (−LGG + HRV and + LGG + HRV groups). The pig body weight at euthanasia did not differ among different treatment groups. Colonic contents and serum samples were collected at euthanasia as previously described [16]. After virulent HRV challenge, rotavirus diarrhea and fecal virus shedding were monitored from PCD 1 to 7 (14).

### 2.4 Microbial community analysis

Colonic contents from the pig large intestine were collected at euthanasia and stored at −80°C. DNA from human stools and pig intestinal contents was extracted with the QIAamp stool mini kit following manufacture’s instructions. The 16S rRNA gene amplicons were generated by PCR with 515 F and barcoded 806R primers [20]. Purified amplicons were sequenced with Illumina MiSeq™.

Sequencing reads were processed with Quantitative Insights Into Microbial Ecology (qiime) [21]. High quality reads with Phred quality score ≥20 (corresponding to an sequencing error rate ≤ 0.01) were clustered into operational taxonomic units (OTUs) with the program uclust[22]. Chimeric sequences were identified with ChimeraSlayer[23] and removed from further analysis. Bacterial taxonomy was assigned by using a naïve Bayes classifier [24] against reference databases and bacterial taxonomy maps at Greengenes [25]. A phylogenetic tree was constructed [26] from PyNAST-aligned sequences representing each OTU. Principle coordinate analysis on stool samples was based on UniFrac distances [27]. Distance-based redundancy analysis for effect of HRV on community structures was performed with the vegan package [28]. Shannon and Simpson diversity indices and a rank abundance curve were both generated with qiime.

The nucleotide sequences have been deposited to MG-RAST [29] with the accession number 4547774.3. Comparison of data with respect to LGG and HRV treatment was done with unpaired t-tests, Mann–Whitney test without assuming normal distributions, One-Way or Two-Way ANOVA.

## 3 Results

**Figure 1:**
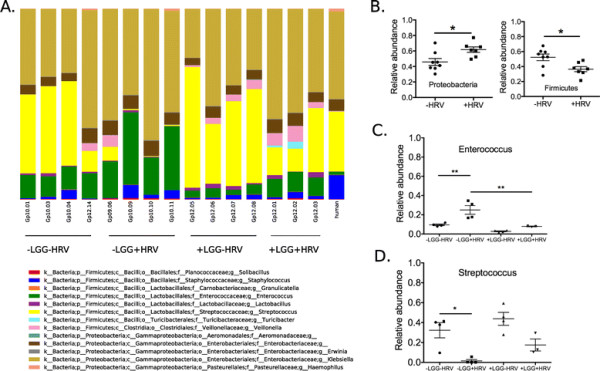
**Bacterial taxonomic summary for the human donor and the recipient gnotobiotic pigs. A**: Taxonomy breakdown at the genus level (for genera collectively accounting for more than 0.5% of total community) for all subjects. Probiotic treatment and virulent human rotavirus (HRV) challenge are designated as ± LGG or ± HRV, respectively. For example, −LGG-HRV means no LGG treatment and no virulent HRV challenge. **B**: HRV infection changes the relative abundance of Proteobacteria and Firmicutes regardless of LGG. Error bars represent standard error of the mean. The *p* values are based on Mann–Whitney test. The statistical significance was the same when we used the unpaired *t*-test to log-transformed data. **C**: Combination of LGG and HRV changes relative abundance of *Enterococcus*. **D**: Combination of LGG and HRV changes relative abundance of *Streptococcus*. The *p* values in **C** and **D** were based on Two-Way ANOVA.

A total of 5,616,353 non-chimeric high quality sequences from feces of the human donor and the recipient pigs were analyzed with QIIME. We analyzed the sequences at the operational taxonomic unit (OTU) level [30]. The recipient pigs carried microbiota that are similar to the human donor’s microbiota (Figure 1A), despite that all pigs had received the attenuated HRV vaccine. Two bacterial phyla, Firmicutes and Proteobacteria, representing over 98% of total bacterial sequences in each subject, dominated microbiota of both the human donor (delivered by C-section) and the recipient pigs. The most abundant genera within Firmicutes were *Streptococcus*, *Enterococcus*, *Veillonella*, and *Staphylococcus* (Figure 1A). The rank abundance curve showed that the gut microbiota of human and pigs had a long tail of rare OTUs (Additional file 1: Figure S1). For example, around 900 OTUs (species rank from 100 to 1000) each accounted only 0.01% (10^−4^) to well below 0.001% (10^−5^) of total bacteria. The human gut microbiota represented by the dashed line appeared to be above the gut microbiota of recipient pigs, suggesting a slightly higher evenness of human microbiota compared with pigs.

We observed a shift in the microbiota composition at phylum, genus, and OTU levels. At the phylum level, the relative abundance of Proteobacteria and Firmicutes in transplanted pigs was affected by HRV challenge (Figure 1B). HRV-challenged pigs had 16% less Firmicutes, and accordingly 16% more Proteobacteria, than non-challenged pigs. The significance of the shift was confirmed by Mann–Whitney test (*P* < 0.05 for both phyla). The phyla Proteobacteria harbor many aerobes and facultative anaerobes, and could serve important roles in removing oxygen diffused from the gut epithelium [31]. We also found that HRV challenge was a factor in changes in overall community structures at the OTU level (Additional file 1: Figure S2), based on results from distance-based redundancy analysis (db-RDA). At the genus level, two abundant genera, *Enterococcus* and *Streptococcus*, were affected by LGG feeding and HRV challenge. In the absence of LGG feeding, HRV-challenged pigs had significantly elevated *Enterococcus* over non-challenged pigs (*P* < 0.01, Figure 1C). This effect was absent for pigs fed with LGG, indicating that LGG prevented HRV’s effect on *Enterococcus*. In HRV-challenged pigs, LGG-treated pigs again had significantly reduced *Enterococcus* (*P* < 0.01, Figure 1C). HRV decreased *Streptococcus* in pigs without LGG feeding (*P* < 0.05, Figure 1D), but the effect disappeared in LGG-fed pigs, suggesting again that LGG prevented microbiota perturbation by rotavirus infection. The combination of LGG and HRV also affected less abundant genera including *Veillonella* and *Aeromonas* (data not shown). Similar to our findings, an increase in Proteobacteria was reported in norovirus-infected humans [32].

**Figure 2:**
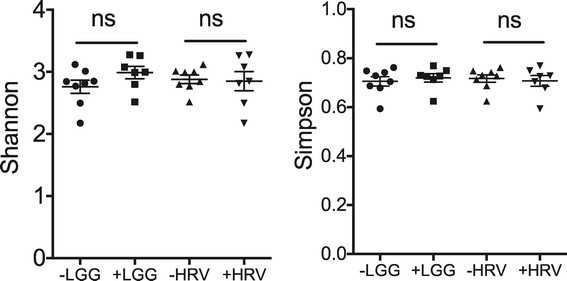
**Shannon (H) and Simpson (E) diversity indices of pig microbiota with respect to LGG and HRV treatment.** Values for the human donor microbiota are: H = 3.017 and E = 0.757. ns: not significant based on unpaired *t*-tests.

We were interested in whether HRV and/or LGG changed overall community richness and evenness. We found that HRV challenge had no significant effect on Shannon or Simpson diversity indices. LGG-treated microbiota appeared to be slightly more diverse than untreated microbiota, but the effect was not significant (Figure 2).

**Figure 3:**
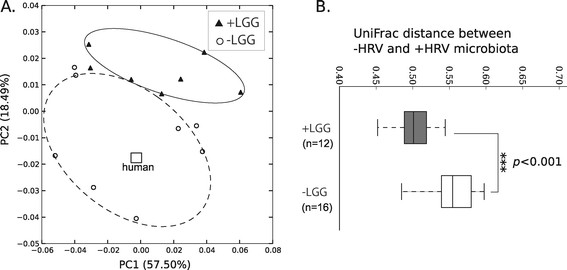
**Phylogenetic dissimilarities among transplanted pigs and the effect of LGG treatment. A**: A principal coordinate (PCoA) analysis of weighted UniFrac distances among all pigs. Only the first two axes (PC1 and PC2) that explain largest variations among samples are plotted. Open circles are pigs receiving no LGG, and filled triangles are pigs treated with LGG. The open square indicates the human microbiota. Significant grouping by LGG was tested by PERMANOVA described in the text. **B**: The UniFrac distances between HRV-challenged pig microbiota versus non-challenged pig microbiota. These distances were divided into two groups according to whether LGG was added. The test of significance was performed using the Mann–Whitney test.

To analyze LGG’s effect on microbial community structures, we performed principal coordinate analysis (PCoA) on weighted UniFrac distances. The results showed that LGG-treated pig microbiota was distinct from those receiving no LGG (Figure 3A), supported by a permutational multivariate analysis (PERMANOVA) with a *p* value of 0.005 at 999 permutations [33]. The human microbiota appeared to cluster closer with –LGG pigs. Figure 3B showed that the extent to which HRV changed microbiota depended on LGG. The HRV-caused microbiota change, measured by UniFrac distances between + HRV and –HRV pigs, was smaller for LGG treated pigs than for no-LGG treated pigs (*p* < 0.001, Figure 3B), suggesting an interaction between LGG and HRV on the microbiota structure. Overall, LGG treatment could resist the change of microbial community structures caused by HRV challenge.

## 4 Discussion

In this study, we demonstrated that human gut microbiota could be transplanted to and colonize gnotobiotic pigs. The resulting “humanized pigs” share the majority of human donor microbiota, albeit the human donor microbiota appeared to be more even than the colonized pig gut microbiota. Virulent rotavirus challenge changed *Enterococcus* and *Streptococcus* abundance in the humanized pig microbiota. Adding probiotic LGG prevented these changes.

The human infant microbiota used in our study was a composite sample from a week-long daily collection of feces. Our intention was to reduce the temporal variations and dynamics known for human microbiota of this age [34],[35]. We acknowledge that effect of inter-personal microbiota variations on colonization of Gn pigs would need to be evaluated through the use of fecal samples from a higher number of infants. Moreover, the inter-pig variations in HGM colonization could be minimized through the use of highly in-bred pigs. Overcoming these limitations will allow us to quantify how stable the microbiota of recipient pigs are.

We chose a C-section delivered HGM donor in this study because of the increasing popularity of this delivery mode. The dominance of Firmicutes and Proteobacteria while lacking Actinobacteria and Bacteroidetes is typical for C-section delivered babies and different from vaginally-delivered babies [36],[37]. In particular, Bifidobacteria, a prominent group in the phylum Actinobacteria, were absent in microbiota from C-section delivered infants, but was found abundant in vaginally-delivered infants [36]. In another high-throughput sequencing study, the mode of delivery was found to be the main determinant of newborn’s microbiota [37]. In that study, C-section delivered babies lacked mothers’ vaginal species within Actinobacteria; Bacteroidetes was found to be mainly in the vaginal delivered babies. The same study also reported that *Staphylococcus* species appeared in C-section delivered babies, which agreed with our results (Figure 1A, *Staphylococcus* in dark blue color).

Previous studies have demonstrated that the oral HRV vaccine does not alter the gut microbiota in older children [38]. This provided rationale for us not to include HMG pigs without an HRV vaccine. All the Gn piglets in our study were vaccinated with attenuated HRV, yet the microbiota in the vaccinated pigs was still significantly altered by virulent HRV **(**Figure 1B and Additional file 1: Figure S2). We speculate that the extent of microbiota alterations caused by virulent HRV would have been larger if the Gn pigs were not vaccinated. This hypothesis warrants further investigation, as most children in developing countries are not vaccinated against HRV. We found LGG could prevent certain changes in microbiota induced by HRV, suggesting potential interacting effects of LGG and HRV on microbiota, although the exact nature of such interactions is unclear.

Szajewska et al. [11] demonstrates that LGG improves HRV-induced diarrhea. That study used subjects 1 month to 18 years old but did not specify the delivery type. It is possible that LGG may not have the same protective effects on C-section delivered infants which contain a different gut microbiota compared with vaginal delivered infants. Although all the pigs studied received the oral attenuated HRV vaccine, the protection against virulent HRV induced diarrhea was only partial [16]. There were no significant differences in protection rate against diarrhea or virus shedding, the severity of diarrhea, or the titer of virus shedding (data not shown); therefore we could not evaluate quantitatively whether the changes in the microbiome correlate with protection against diarrhea or virus shedding. Fifty percent (2/4) of the pigs in the –LGG + HRV group were protected from infection upon virulent HRV challenge. There is no apparent difference in the abundant taxa between protected (Gp09.06 and Gp10.09) versus unprotected (Gp10.10 and Gp10.11) pigs (Figure 1A, group “-LGG + HRV”). However, the low abundance bacterial taxa (Additional file 1: Figure S3), which accounted for less than 0.5% of the microbiota, showed that one of the two protected pigs (Gp09.06) harbored unique bacteria such as Ruminococcaceae. The other protected pig (Gp10.09) shared similar low-abundance taxa with unprotected pigs. Further studies with more animals would be needed to identify rare taxa potentially associated with viral protection.

Several relevant issues that were not resolved in the present study will be addressed in future studies. These include (a) the effect of vaccination on the gut microbiota. (b) Microbiota in human infants varies among individuals and changes during the first few months of life, especially at weaning. Our study used HGM from only one C-section delivered newborn; a HGM mixture from multiple older children matching the age of rotavirus vaccination (e.g., 2 to 6 months) will be a better model for vaccine evaluation. Additional data from both C-section-derived and conventional birth-derived infants would be highly desirable to further evaluation the HGM pig model. (c) The LGG feeding in this study did not significantly improve the protection conferred by the rotavirus vaccine. Changes in the microbiome due to LGG feeding were not associated with a change in the protective efficacy of the vaccine. Adjustment of the dose and dosing regimen of LGG may be needed in order for LGG to exert its adjuvant effect on the rotavirus vaccine. The microbiome structure and composition that may favor stronger immunogenicity and protective efficacy of rotavirus vaccines require further studies to identify.

In conclusion, the HGM-transplanted gnotobiotic pig presents a useful model for testing interventions using probiotics and vaccines to prevent or treat infantile diarrhea and improve enteric health and immunity. Future study using a mixture of feces from multiple older children that match the age of rotavirus vaccination will improve this model for vaccine evaluation.

## Competing interests

The authors declare that they have no competing interests.

## Authors' contributions

LY designed the study. HW, KW, GL, XY, JK, EGR carried out the experiments, HZ, AD, RS performed data analysis. HZ, MS, LY wrote the manuscript. All authors read and approved the final manuscript.

## Additional file

## Supplementary Material

Additional file 1: Figure S1. Relative abundance of OTUs plotted against OTU rank. From left to right, high-ranking OTU with high abundance towards low-ranking OTU with low abundance. **Figure S2.** Distance-based redundancy analysis (db-RDA) of pig microbiota in response to HRV challenge. CAP1 and MDS1 are constrained and unconstrained axes, respectively. HRV-challenged pigs are indicated by ovals, and LGG supplemented pigs are indicated by underlines. The arrow indicates source of variation explained by HRV. **Figure S3.** Rare taxa (OTUs <0.5% of total bacteria) in the -LGG+HRV group. Gp09.06 and Gp 10.09 were protected from infection by attenuated HRV vaccines. Gp 10.10 and Gp10.11 were unprotected. Click here for file
