# Supplementary material for: Probiotics and virulent human rotavirus modulate the transplanted human gut microbiota in gnotobiotic pigs
Source: Gut Pathog. 2014 Sep 9;6:39. doi: 10.1186/s13099-014-0039-8 (PMC4209515; doi:10.1186/s13099-014-0039-8)
Supplement: Additional file 1: Figure S1. — Relative abundance of OTUs plotted against OTU rank. From left to right, high-ranking OTU with high abundance towards low-ranking OTU with low abundance. Figure S2. Distance-based redundancy analysis (db-RDA) of pig microbiota in response to HRV challenge. CAP1 and MDS1 are constrained and unconstrained axes, respectively. HRV-challenged pigs are indicated by ovals, and LGG supplemented pigs are indicated by underlines. The arrow indicates source of variation explained by HRV. Figure S3. Rare taxa (OTUs <0.5% of total bacteria) in the -LGG+HRV group. Gp09.06 and Gp 10.09 were protected from infection by attenuated HRV vaccines. Gp 10.10 and Gp10.11 were unprotected. [file s13099-014-0039-8-S1.pdf]

Supplementary Materials

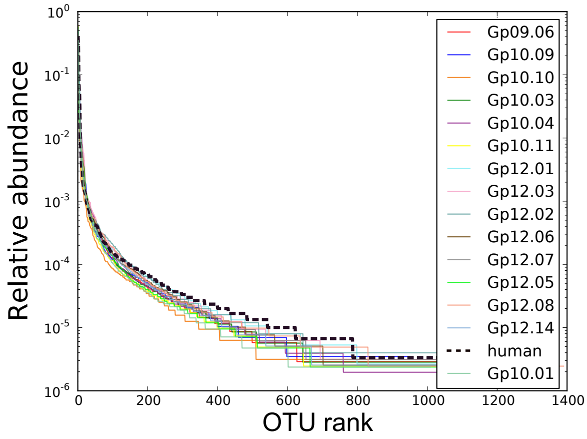

Fig. S1. Relative abundance of OTUs plotted against OTU rank. From left to right, high-ranking OTU with high abundance towards low-ranking OTU with low abundance.

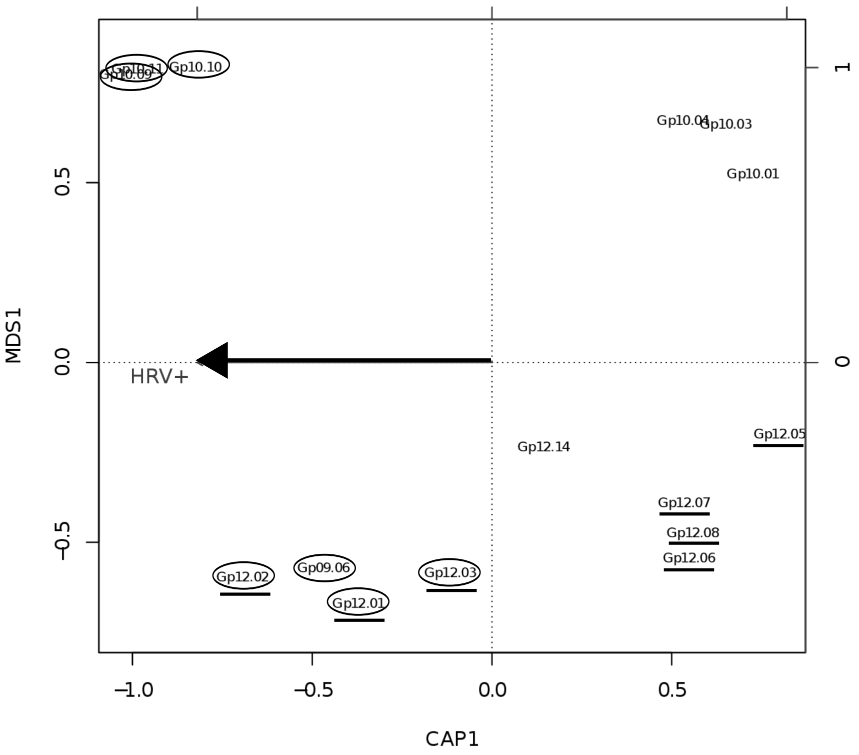

Fig. S2. Distance-based redundancy analysis (db-RDA) of pig microbiota in response to HRV challenge. CAP1 and MDS1 are constrained and unconstrained axes, respectively. HRV-challenged pigs are indicated by ovals, and LGG supplemented pigs are indicated by underlines. The arrow indicates source of variation explained by HRV.

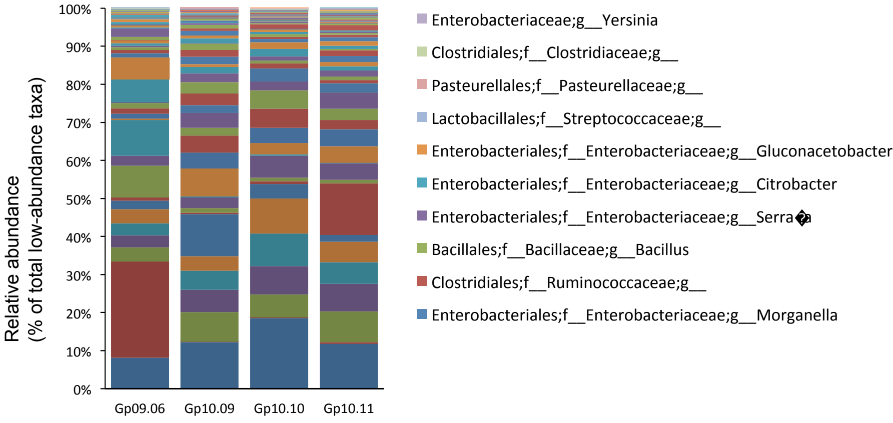

Fig. S3. Rare taxa (OTUs <0.5% of total bacteria) in the -LGG+HRV group. Gp09.06 and Gp 10.09 were protected from infection by attenuated HRV vaccines. Gp 10.10 and Gp10.11 were unprotected.
